# Supplementary material for: Lagged Association between Climate Variables and Hospital Admissions for Pneumonia in South Africa
Source: Int J Environ Res Public Health. 2021 Jun 8;18(12):6191. doi: 10.3390/ijerph18126191 (PMC8228646; doi:10.3390/ijerph18126191)
Supplement: Supplementary file 1 [file ijerph-18-06191-s001.zip › ijerph-1247111-supplementary.pdf]

Supplementary material: Lagged association between climate variables and hospital admissions for pneumonia admissions in South Africa

Model Checking

There does not appear to be any long-term trend in the residuals (Figure S4), though the variance in residuals is lower towards the end of 2013, perhaps due to very low hospital admission counts during this time which may have been due to missing data.

There is also no obvious relationship between residuals and mean daily temperature, daily temperature range or day of the week, indicating that we have suitably accounted for these variables (Figure S5). However, there is a slight trend for residual deviances to increase at very high relative humidity values. Given the scale boundaries (% relative humidity bounded by 0 and 100) and the relatively small number of observations with very high humidity it is challenging to fully model the relationship here. Scale transformation did not affect the residuals and we therefore leave relative humidity on a % scale for interpretability.

Finally, we show the partial autocorrelation between neighboring days to ensure that the lag has been properly accounted for (Figure S6). Whilst there is slightly higher partial autocorrelation for some lags, this has generally been kept reasonably low by the inclusion of linear terms for lagged admission counts over the preceding 3 days.

Sensitivity Analyses

Substituting mean daily temperature with minimum/maximum daily temperature

No significant association was identified between minimum daily temperature and pneumonia admissions (Figure S7). However, there was a strong association between maximum daily temperature and pneumonia admissions, with higher maximum temperatures (>34°C) associated with lower admissions across the whole 21-day lag period (Figure S8). This supports (and may to some degree drive) the association between higher mean daily temperature and lower pneumonia admissions.

Investigating modelling lagged exposure-response association using natural cubic splines with different numbers of knots

No meaningful changes in cumulative lagged exposure relationships were found when using different numbers of knots for the natural cubic spline functions used to model either the exposure-response relationships or the lagged relationships (Figures S9-S11). The biggest changes were to the 95%CIs of predictions due to the additional parameters in the model when functions with more knots are fitted.

Supplementary figures

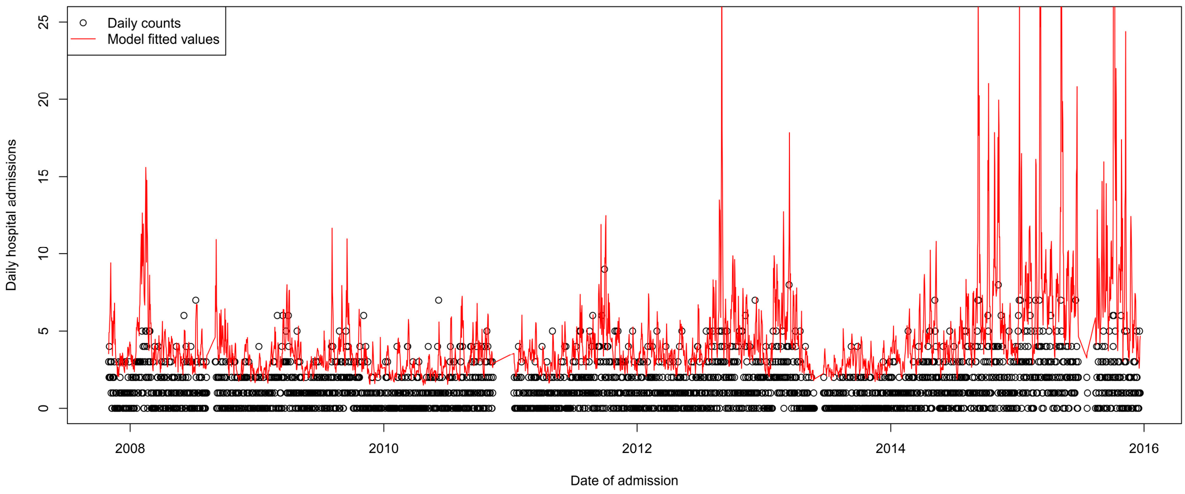

Figure S1: Daily pneumonia hospital admissions (black circles) with model fitted values (red line).

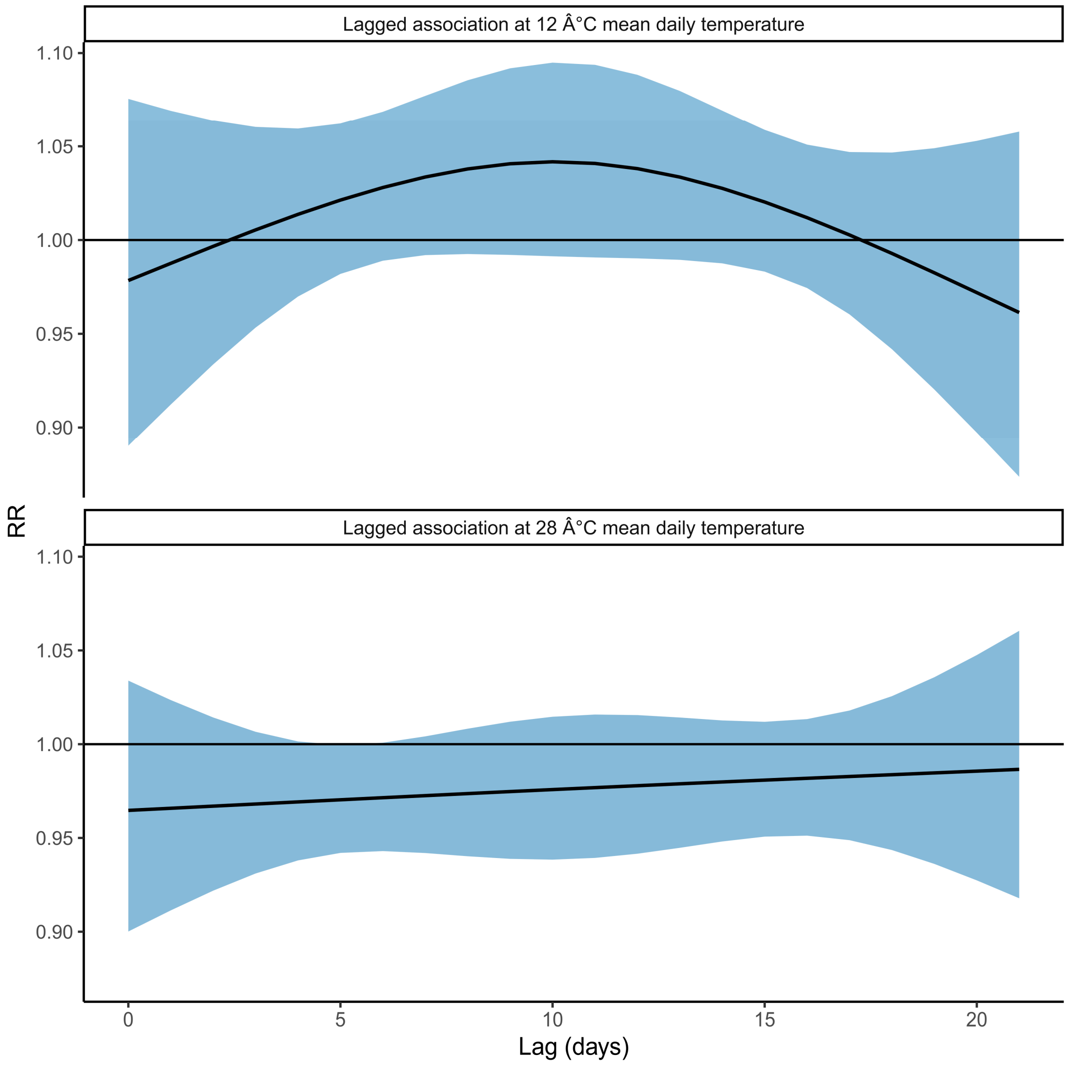

Figure S2: The association between mean daily temperature and pneumonia hospital admissions across different lag durations at 12 and 28°C. The solid curves are the predicted relative rates (RR), and the shaded regions are the 95%CI. The median mean daily temperature (21°C) is used as the reference value against which the RRs are compared.

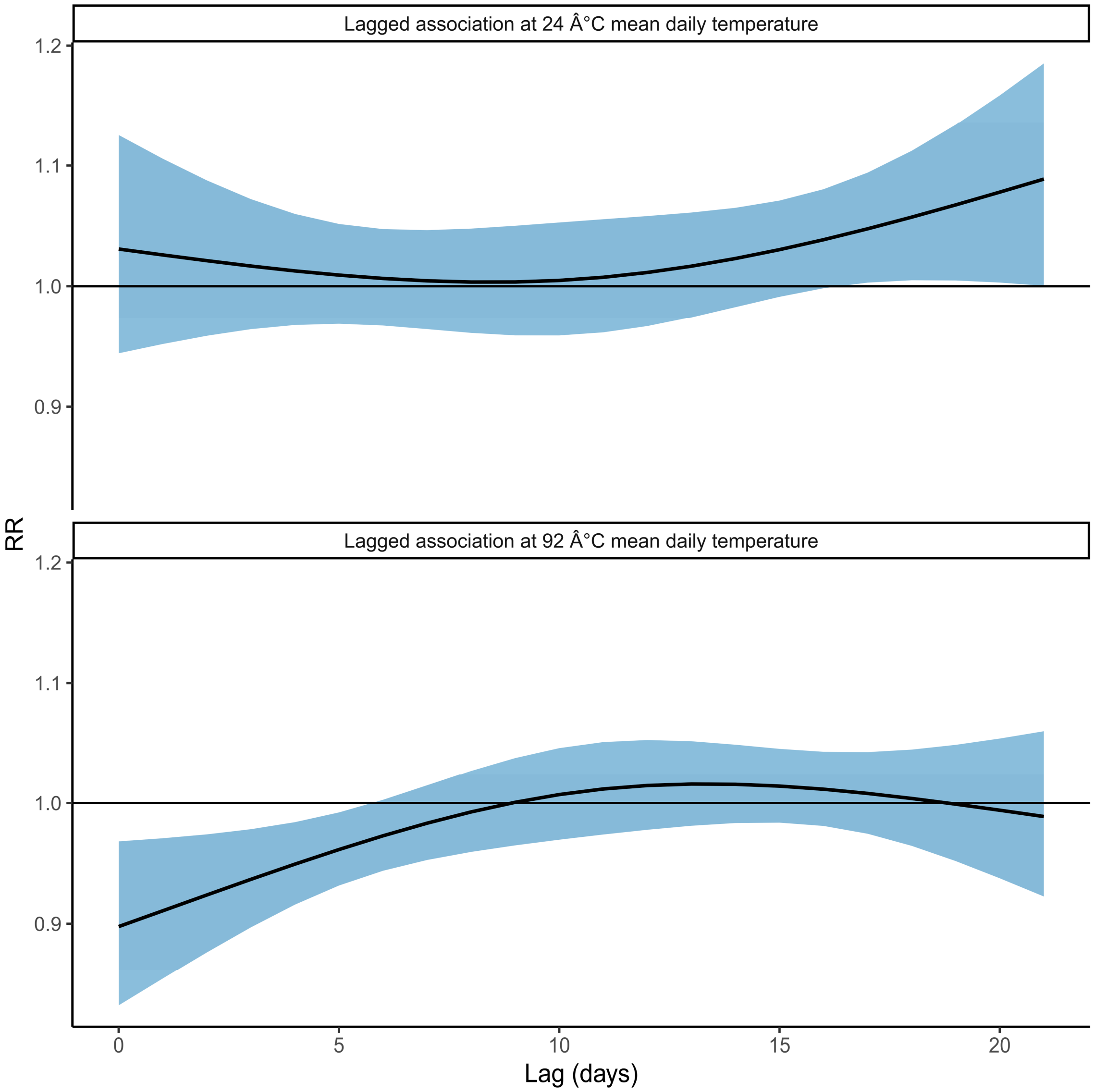

Figure S3: The association between relative humidity and pneumonia hospital admissions across different lag durations at 24 and 92% relative humidity. The solid curves are the predicted relative rates (RR), and the shaded regions are the 95% CI. The median relative humidity (67%) is used as the reference value against which the RRs are compared.

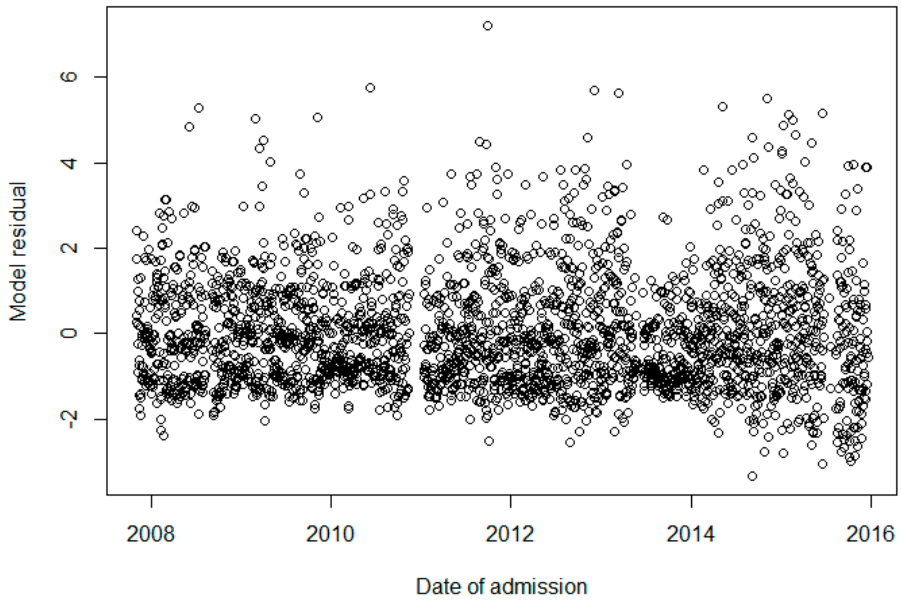

Figure S4: Model residual deviances plotted by date of admission.

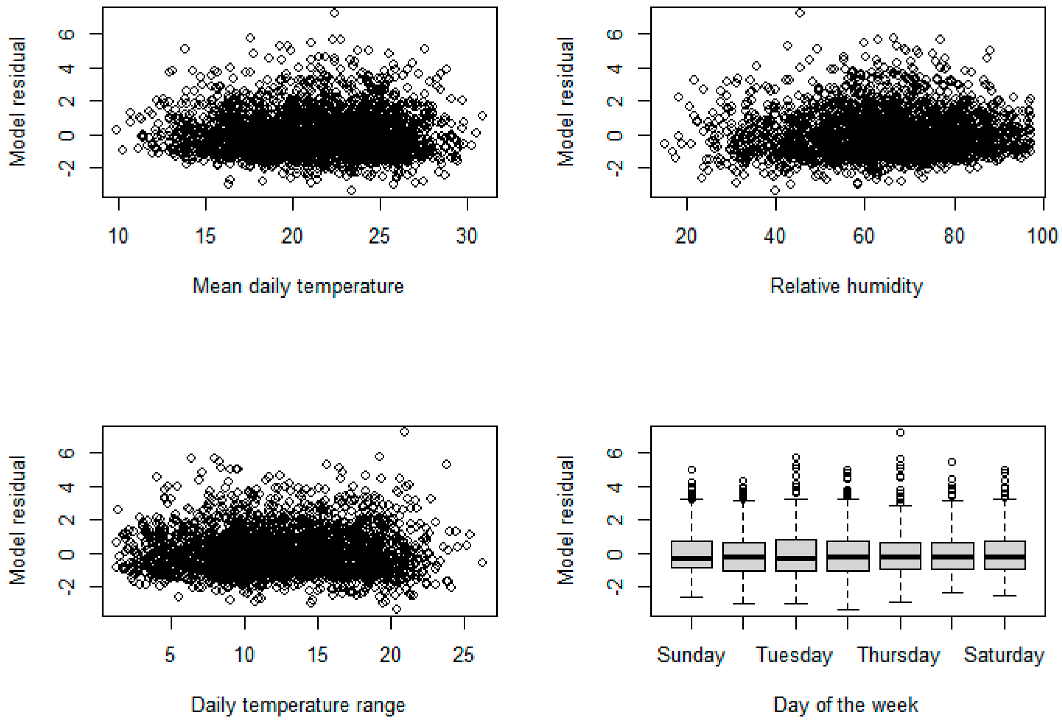

Figure S5: Model residual deviances plotted against model predictors for mean daily temperature, relative humidity, daily temperature range, and day of the week.

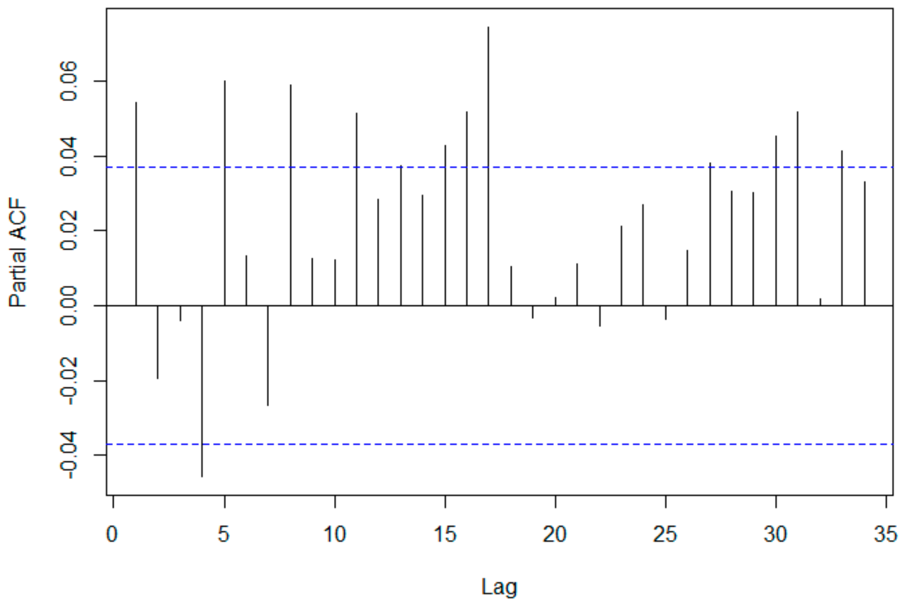

Figure S6: Partial autocorrelation of model residuals between neighboring days separated by different lags.

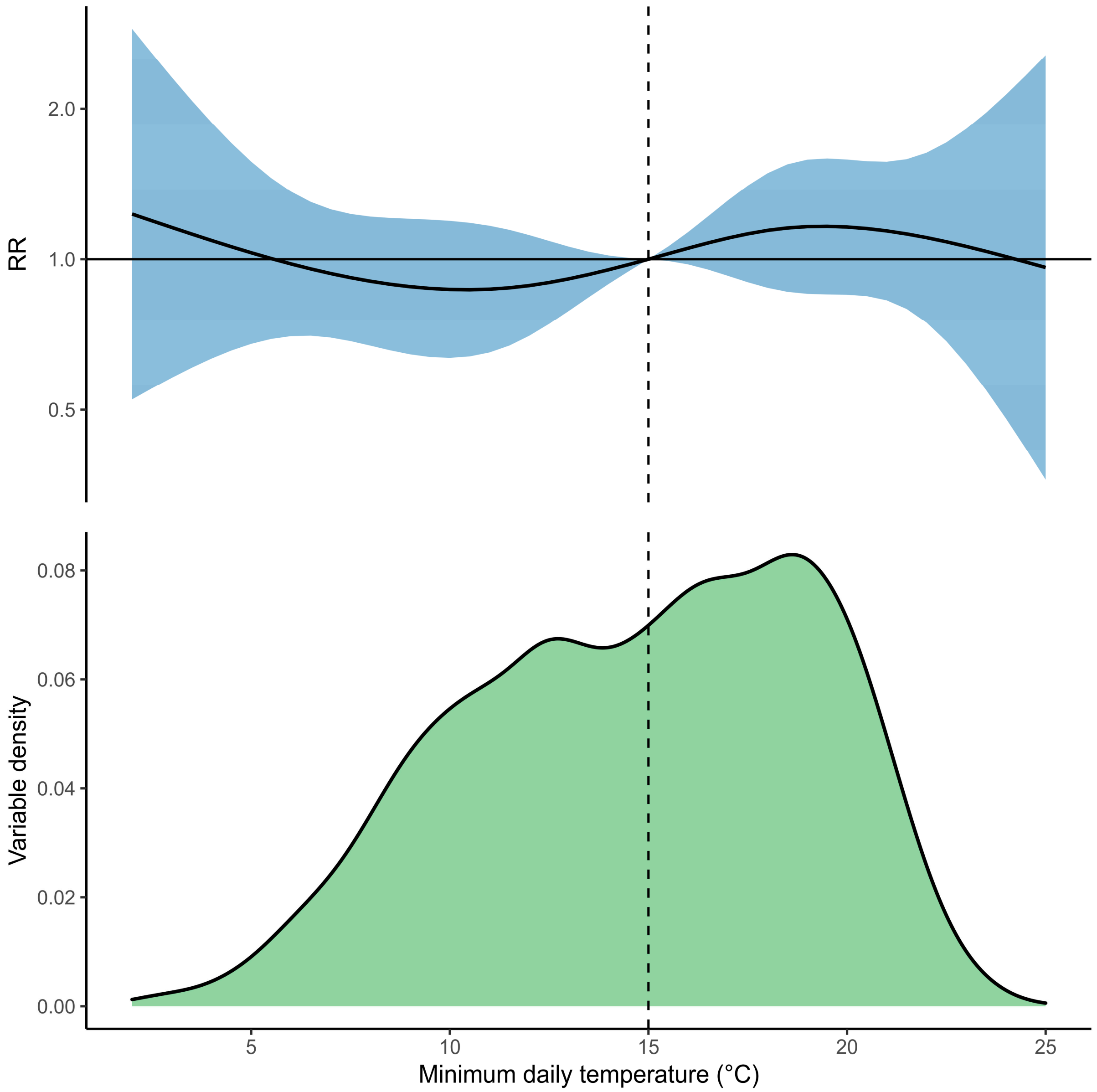

Figure S7: The top panel shows the cumulative association between minimum daily temperature and pneumonia hospital admissions across a 21-day lag period. The solid curve is the predicted relative rates (RR), and the shaded region is the 95% CI. The lower panel shows the distribution of minimum daily temperature within the iDEWS dataset. The median minimum daily temperature (15°C) is indicated by the dashed vertical line and is used as the reference value against which the RR for other temperatures is compared.

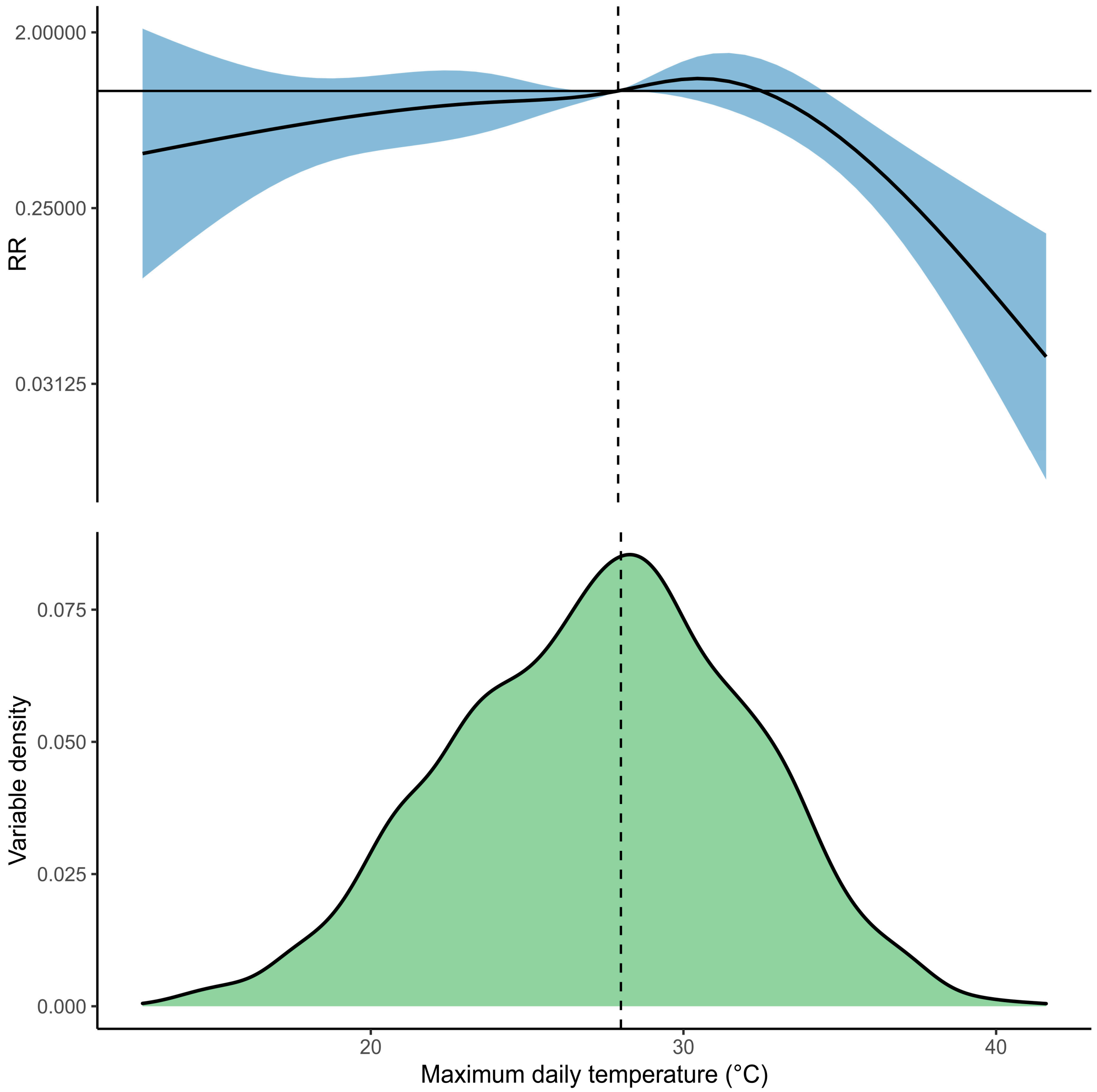

Figure S8: The top panel shows the cumulative association between maximum daily temperature and pneumonia hospital admissions across a 21-day lag period. The solid curve is the predicted relative rates (RR), and the shaded region is the 95% CI. The lower panel shows the distribution of maximum daily temperature within the iDEWS dataset. The median maximum daily temperature (28°C) is indicated by the dashed vertical line and is used as the reference value against which the RR for other temperatures is compared.

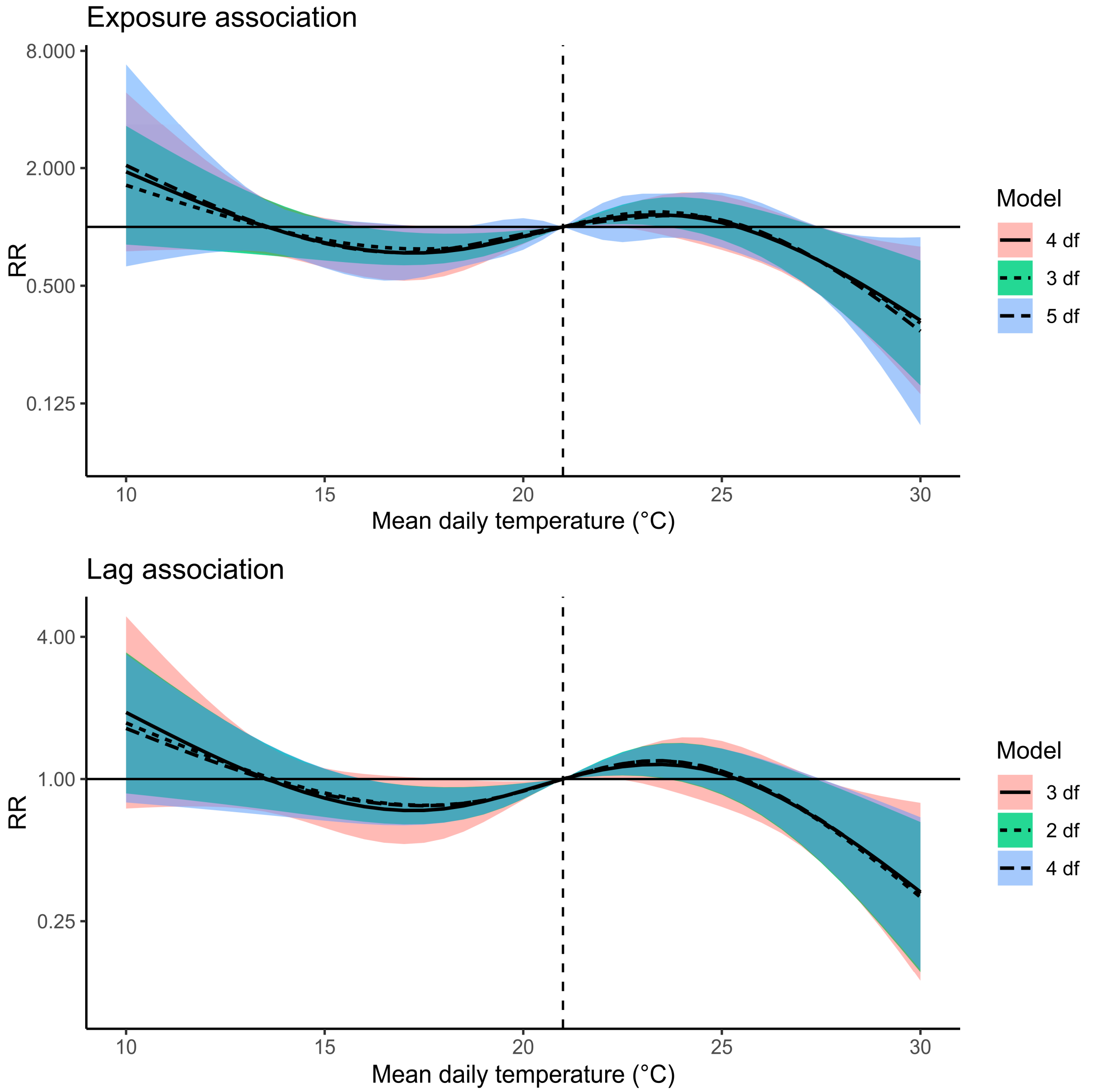

Figure S9: Cumulative associations between mean daily temperature and pneumonia hospital admissions across a 21-day lag period compared to the median mean daily temperature (21°C – shown by the vertical dashed line) for models with different degrees of freedom (df = number of internal knots + 1) used to fit the natural cubic spline function for the exposure-response association (top panel) and the lagged association (lower panel). Predictions from the selected model are shown by the solid line with the red shaded region representing the 95% CI.

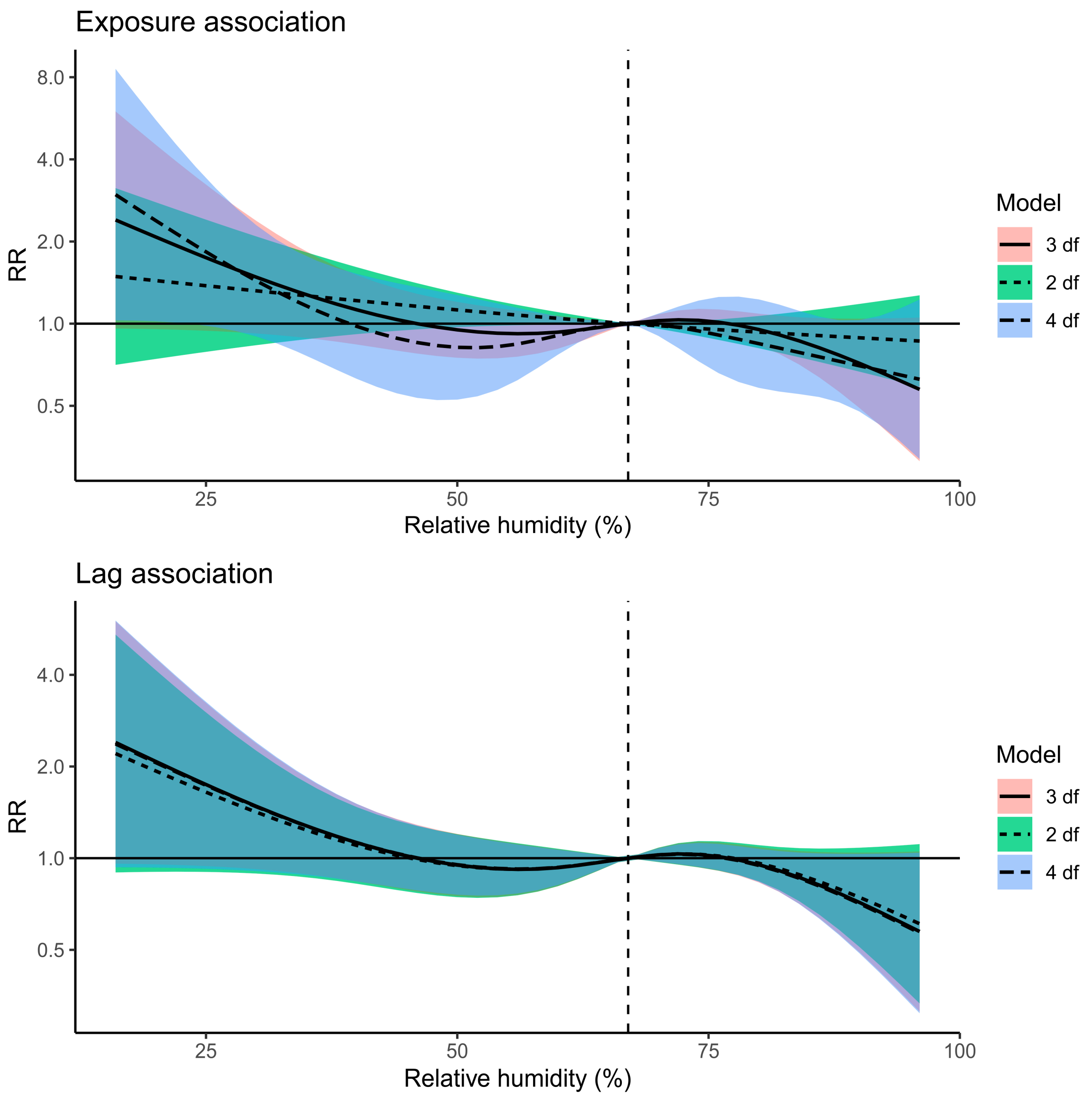

Figure S10: Cumulative associations between relative humidity and pneumonia hospital admissions across a 21-day lag period compared to the median relative humidity (67% – shown by the vertical dashed line) for models with different degrees of freedom (df = number of internal knots + 1) used to fit the natural cubic spline function for the exposure-response association (top panel) and the lagged association (lower panel). Predictions from the selected model are shown by the solid line with the red shaded region representing the 95%CI.

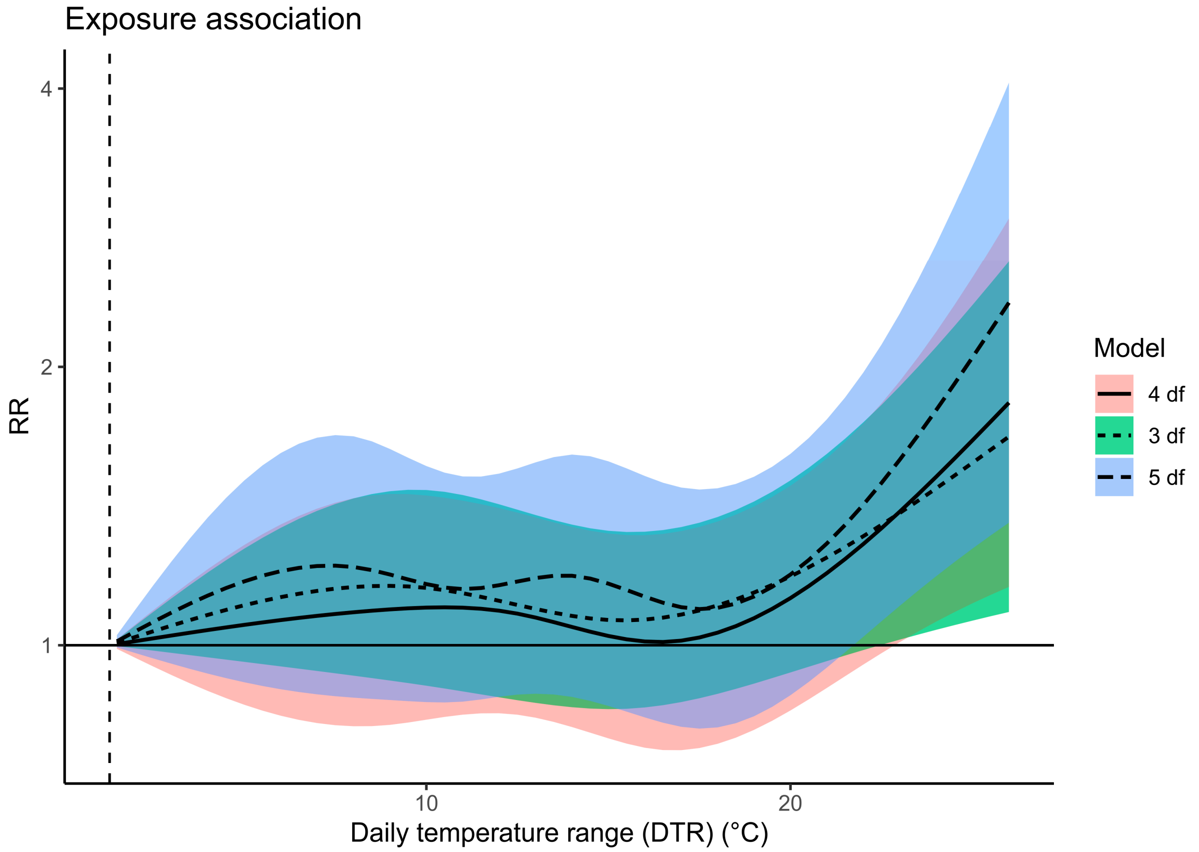

Figure S11: Cumulative associations between daily temperature range (DTR) and pneumonia hospital admissions across a 21-day lag period compared to the optimal minimum DTR (1.3°C – shown by the vertical dashed line) for models with different degrees of freedom (df = number of internal knots + 1) used to fit the natural cubic spline function for the exposure-response association. Predictions from the selected model are shown by the solid line with the red shaded region representing the 95% CI.
